# Integrated Downstream Analysis and Epidemiological Modelling of Hantavirus Infection: From Host Transcriptomics to Transmission Dynamics

**DOI:** 10.3390/pathogens15060601

**Published:** 2026-06-03

**Authors:** Pietro Hiram Guzzi, Francesco Branda, Fabio Scarpa, Giancarlo Ceccarelli, Massimo Ciccozzi, Federico Manuel Giorgi, Pierangelo Veltri

**Affiliations:** 1 Department of Surgical and Medical Sciences, University “Magna Graecia” of Catanzaro, 88100 Catanzaro, Italy; hguzzi@unicz.it; 2 Unit of Medical Statistics and Molecular Epidemiology, Università Campus Bio-Medico di Roma, Via Álvaro del Portillo 21, 00128 Rome, Italy; m.ciccozzi@unicampus.it; 3 Department of Biomedical Sciences, University of Sassari, Viale San Pietro 43, 07100 Sassari, Italy; fscarpa@uniss.it; 4 Department of Public Health and Infectious Diseases, University Hospital Policlinico Umberto I, Sapienza University of Rome, 00185 Rome, Italy; giancarlo.ceccarelli@uniroma1.it; 5 Department of Pharmacy and Biotechnology, University of Bologna, 40126 Bologna, Italy; federico.giorgi@unibo.it; 6 DIMES, University of Calabria, 88100 Rende, Italy; pierangelo.veltri@unical.it

**Keywords:** hantavirus, HFRS, HCPS, transcriptomics, interferon signalling, protein–protein interaction network, regulatory hubs, SEIRD model, zoonosis, rodent-to-human transmission

## Abstract

Hantaviruses are emerging zoonotic pathogens responsible for two severe clinical syndromes: (i) haemorrhagic fever with renal syndrome (HFRS) and (ii) hantavirus cardiopulmonary syndrome (HCPS), collectively causing more than 200,000 human cases annually worldwide. Despite their public-health importance, the molecular mechanisms governing the host response and the population-level dynamics of rodent-to-human spillover remain incompletely characterised. The timeliness of this framework is underscored by the April–May 2026 outbreak of Andes orthohantavirus aboard the MV Hondius cruise ship, the first such cluster in a maritime setting, with three deaths reported across multiple countries. This event revealed critical gaps in existing models that treat humans solely as dead-end spillover hosts. Our coupled Susceptible-Exposed-Infectious-Recovered-Dead (SEIRD) model assumes no human-to-human transmission and is therefore designed for hantavirus strains where spillover does not lead to secondary human cases, specifically Hantaan virus (HTNV), Puumala virus (PUUV), Sin Nombre virus (SNV), and Dobrava-Belgrade virus (DOBV). The Andes virus (ANDV) outbreak aboard the MV Hondius is used as a real-world case study to assess the boundaries of our model and to motivate future extensions, not as a direct validation target for its quantitative predictions. Here, we present an integrated computational study combining three complementary analyses. First, we performed a preliminary phylogenetic analysis of the viral sequence, identifying *Orthohantavirus andesense* as the likely etiological agent responsible for the vessel-associated outbreak. Second, we carried out a downstream transcriptomic analysis of Hantaan virus (HTNV)-infected human umbilical vein endothelial cells (HUVECs), using publicly available RNA-seq data (GEO accession GSE133751, n=3 per group). This analysis identified 184 upregulated and 19 downregulated genes, highlighting a transcriptional response dominated by interferon-stimulated genes (ISGs), including *CXCL10*, *CXCL11*, *MX2*, *DDX58*, *IRF7*, *STAT1*, *OASL*, and *CMPK2*. We then constructed a protein–protein interaction (PPI) network using STRING, comprising 176 nodes and 3210 edges, and applied a composite network centrality score to rank putative regulatory hubs. This analysis identified *ISG15*, *IRF1*, *CXCL10*, *STAT1*, and *DDX58* as the most central nodes. Pathway enrichment analysis confirmed a strong activation of interferon signalling (Reactome, $p=1.3\times 10^{-63}$), antiviral defence mechanisms (Gene Ontology, $p=3.8\times 10^{-58}$), and NF-κB-related pathways, together with a concurrent suppression of ribosomal translation. Finally, we developed a coupled SEIRD epidemiological model that explicitly represents rodent-to-rodent and rodent-to-human transmission with logistic rodent population growth. Preliminary simulation analysis demonstrates that reducing human exposure to rodent excreta is substantially more effective than rodent population control alone for reducing human disease burden, and that rodent control in isolation can paradoxically increase human cases through a dilution-like effect. The integrated framework provides molecular and epidemiological insights relevant to hantavirus surveillance, therapeutic target identification, and public-health intervention design.

## 1. Introduction

Hantaviruses (family *Hantaviridae*, order *Bunyavirales*) are enveloped, negative-sense single-stranded RNA viruses. Orthohantaviruses have a tripartite genome of approximately 11.8–13.8 kb, comprising small (S, ∼1.7–3 kb), medium (M, ∼3.2–4.9 kb), and large (L, ∼6.5–6.8 kb) RNA segments that encode the nucleocapsid protein (NP), the glycoprotein precursor (GPC), and the RNA-dependent RNA polymerase (RdRp), respectively [1]. Unlike most other bunyaviruses, hantaviruses are not arthropod-borne; instead, they are maintained in specific rodent reservoir hosts and are transmitted to humans primarily through inhalation of aerosolised excreta (urine, faeces, and saliva) from infected rodents [2].

Hantavirus infections cause two distinct clinical syndromes. HFRS, prevalent across Eurasia, is characterised by acute kidney injury, thrombocytopenia, and haemorrhage, with case fatality rates (CFRs) ranging from less than 1% for Puumala virus (PUUV) to 5–15% for Hantaan virus (HTNV) and Dobrava-Belgrade virus (DOBV) [1]. HCPS, predominant in the Americas, manifests as acute respiratory distress syndrome with a CFR of 30–40% for Sin Nombre virus (SNV) and Andes virus (ANDV) [1]. Globally, an estimated 200,000 cases are reported annually, though substantial under-reporting is suspected [3]. Andes virus is unique among hantaviruses in supporting limited human-to-human transmission, raising concerns about pandemic potential [4]. The principal rodent reservoirs are geographically partitioned: HTNV is maintained in the striped field mouse (*Apodemus agrarius*) in East Asia; PUUV in the bank vole (*Myodes glareolus*) in Europe; SNV in the deer mouse (*Peromyscus maniculatus*) in North America; and ANDV in the long-tailed pygmy rice rat (*Oligoryzomys longicaudatus*) in South America [1,2]. Rodent population dynamics, driven by climate, land use, and food availability, are major determinants of outbreak risk [2,5].

Among all known hantaviruses, Andes orthohantavirus (ANDV), maintained primarily by the long-tailed pygmy rice rat (*Oligoryzomys longicaudatus*) across the Andean regions of Argentina and Chile, occupies a distinctive epidemiological position: it is the sole hantavirus for which person-to-person transmission has been conclusively demonstrated through both epidemiological investigation and whole-genome sequencing evidence [6,7]. The first direct genetic proof of human-to-human spread was documented during a 1996 cluster in El Bolsón, Argentina, where phylogenetic analysis of the S and M genome segments revealed a single shared viral haplotype (Epilink/96) across all epidemiologically linked cases [8]. Subsequent investigations established that transmission occurs predominantly during the prodromal phase of illness, requires close and sustained contact, and proceeds via respiratory secretions and saliva, with viral antigen detected in alveolar epithelial cells, macrophages, and submandibular salivary gland secretory cells [9]. A landmark outbreak in Epuyén, Chubut Province, Argentina (November 2018–February 2019) resulted in 34 confirmed infections and 11 deaths, with an estimated reproductive number of $R_{0}=2.12$ prior to the enforcement of isolation measures, falling to 0.96 thereafter—demonstrating that ANDV can sustain self-amplifying chains of human transmission in the absence of timely public health intervention [10]. Genomic analyses of the outbreak strain (ARG-Epuén) subsequently identified specific amino acid substitutions in the nucleocapsid protein, glycoprotein, and small non-structural protein that are associated with enhanced human transmissibility [11,12], raising the prospect that ANDV could evolve toward more efficient human-to-human spread, warranting close genomic surveillance of circulating lineages.

The pandemic potential of ANDV was brought into sharp international focus in April–May 2026 by an unprecedented outbreak aboard the Dutch expedition cruise ship MV Hondius. The vessel departed Ushuaia, Argentina, on 1 April 2026, carrying 147 passengers and crew on a polar voyage to Antarctica and across the South Atlantic Ocean [13]. Illness onset among passengers began as early as 6 April, characterised by fever, gastrointestinal symptoms, and rapid progression to pneumonia and acute respiratory distress syndrome, with three deaths confirmed by 9 May 2026 and six laboratory-confirmed cases of ANDV infection identified across multiple countries [13,14]. South African laboratory testing confirmed the Andes strain in evacuated passengers, and the US Centers for Disease Control and Prevention classified the event as a Level 3 emergency response [14]. Critically, the MV Hondius event represents the first documented ANDV cluster in a cruise ship setting and provides compelling epidemiological evidence of sustained person-to-person transmission in a confined, internationally mobile environment—a scenario with direct implications for global health security [13,14]. This outbreak underscores three key gaps that the present study addresses: the need for a mechanistic understanding of the molecular host response to hantavirus infection at the transcriptional network level, the importance of quantifying the conditions under which human-to-human transmission can become self-sustaining within the epidemiological model, and the urgency of identifying network-level regulatory hubs that could serve as targets for antiviral countermeasures applicable across pathogenic hantavirus strains.

At the molecular level, hantaviruses infect endothelial cells, macrophages, and dendritic cells, exploiting $\beta_{3}$ integrins as entry receptors. The resulting innate immune response is characterised by robust induction of type I interferons (IFN-α/β) and interferon-stimulated genes (ISGs), yet pathogenic hantaviruses have evolved mechanisms to delay and partially suppress this response [15]. The balance between viral immune evasion and host antiviral defence determines disease severity and outcome. Despite considerable progress, the transcriptional regulatory architecture governing the host response to hantavirus infection—and in particular the identity of master regulatory hubs—has not been systematically characterised using network-based approaches.

Epidemiological modelling of hantavirus transmission has been pursued using compartmental modelling  [16,17,18]. These models capture the essential dynamics of rodent-to-rodent transmission and spillover to humans, but few studies have integrated molecular findings with epidemiological projections to provide a unified view of hantavirus biology. In this work, we address this gap by presenting an integrated computational framework that combines: (i) downstream analysis of publicly available transcriptomic data from HTNV-infected HUVECs; (ii) PPI network construction and centrality-based identification of regulatory hubs; and (iii) a coupled SEIRD (Susceptible, Exposed, Infected, Recovered, Diseased) epidemiological model with logistic rodent population dynamics and explicit rodent-to-human spillover. Our approach is inspired by and extends previous network-based analyses of viral infections, including master regulator identification in SARS-CoV-2 [19], graph diffusion frameworks for network analysis [20], and graph-based epidemic modelling of West Nile virus [21]. Together, these analyses provide a multi-scale view of hantavirus infection that bridges molecular mechanisms and population dynamics.

## 2. Background

### 2.1. Hantavirus Biology and Epidemiology

The hantavirus genome encodes three structural proteins. The NP (S segment) encapsidates the viral RNA and plays a central role in innate immune evasion by sequestering double-stranded RNA intermediates [22]. The glycoproteins Gn and Gc (M segment) mediate receptor binding and membrane fusion; Gc contains a class II fusion peptide structurally homologous to flavivirus E proteins. The RdRp (L segment) performs genome replication and transcription in the cytoplasm.

Transmission occurs almost exclusively through inhalation of infectious aerosols, although direct contact with rodent excreta and, rarely, rodent bites have been documented [1]. The incubation period ranges from 1 to 8 weeks. HFRS progresses through febrile, hypotensive, oliguric, diuretic, and convalescent phases;   HCPS is characterised by a prodromal febrile illness followed by rapid cardiopulmonary deterioration [1].  

HFRS progresses through five clinically distinct phases: febrile (days 1–5, abrupt-onset fever, headache, myalgia, facial flushing), hypotensive (days 4–6, thrombocytopenia, proteinuria, haemorrhagic manifestations including petechiae and conjunctival haemorrhage), oliguric (days 5–8, acute kidney injury with rising creatinine, electrolyte imbalances, risk of pulmonary oedema), diuretic (days 9–14, polyuric recovery with risk of dehydration and electrolyte disturbance), and convalescent (weeks to months) [23]. Laboratory hallmarks include thrombocytopenia (nadir $<50\times 10^{9}$ L^−1^ in severe HTNV infection), elevated serum creatinine, haematuria, proteinuria, and elevated LDH [24,25,26].

HCPS, in contrast, presents with a prodromal phase of 3–6 days of fever, myalgia, and gastrointestinal symptoms, followed by abrupt cardiopulmonary deterioration characterised by non-cardiogenic pulmonary oedema, hypoxaemia, and cardiogenic shock due to myocardial depression—the latter distinguishing HCPS from other causes of ARDS [27]. Laboratory findings include haemoconcentration, thrombocytopenia, elevated lactate, and a characteristic peripheral blood smear showing immunoblasts [27].

No licensed antiviral therapy exists; treatment is supportive, with ribavirin showing modest benefit in early HFRS [1].

Seroprevalence studies indicate that subclinical infection is common, with population seroprevalence ranging from 1% to 10% in endemic areas [3]. Outbreaks are strongly seasonal and correlate with rodent population peaks driven by mast years (beech and oak seed production) and climate anomalies [2,5].

### 2.2. Host Immune Response to Hantavirus

Hantavirus infection triggers innate immune sensing through cytoplasmic pattern recognition receptors. The viral RNA is detected by RIG-I (encoded by *DDX58*) and MDA5 (encoded by *IFIH1*), which signal through MAVS to activate IRF3 and IRF7, driving IFN-β production [15]. IFN-β signals in an autocrine and paracrine manner through the IFNAR1/IFNAR2 receptor complex, activating the JAK1/TYK2–STAT1/STAT2–IRF9 (ISGF3) transcription factor complex, which induces hundreds of ISGs including *MX1*, *MX2*, *OAS1-3*, *OASL*, *ISG15*, *IFIT1-3*, and *TRIM22* [28].

Pathogenic hantaviruses (HTNV, SNV, ANDV) delay IFN induction relative to non-pathogenic strains (e.g., Prospect Hill virus), partly through NP-mediated suppression of RIG-I signalling [22]. NF-κB activation drives pro-inflammatory cytokine production (TNF-α, IL-6, CXCL10), contributing to the cytokine storm and vascular permeability that underlie disease pathology [28]. Endothelial cell tropism is central to pathogenesis: hantavirus-infected endothelial cells exhibit increased permeability without cytopathic effect, a hallmark of both HFRS and HCPS [1]. The clinical severity of both HFRS and HCPS correlates with the magnitude of the host immune response rather than with direct viral cytopathic effect. Elevated plasma levels of CXCL10 (IP-10), TNF-α, and IL-6—genes identified as upregulated in our transcriptomic analysis—have been associated with disease severity and poor outcome in both syndromes [1,29]. Endothelial hyperpermeability, the central pathological event, results from immune-mediated disruption of VE-cadherin junctions rather than endothelial cell lysis, explaining the absence of cytopathic effect in infected HUVECs [1]. Thrombocytopenia arises from platelet consumption at sites of vascular injury and impaired megakaryocyte function [25,30]. In HCPS, myocardial depression is driven in part by TNF-α and IL-6-mediated cardiomyocyte dysfunction, a mechanism with direct implications for intensive care management [1,29].

### 2.3. Computational Approaches to Viral Infection Analysis

Network medicine has emerged as a powerful framework for understanding viral infections at the systems level. PPI networks, constructed from databases such as STRING [31] and BioGRID, enable identification of central regulatory nodes (hubs) that coordinate the host response. Centrality metrics—degree, betweenness, and closeness—have been used to prioritise therapeutic targets and identify master regulators in diverse viral infections [19]. In the context of SARS-CoV-2, Guzzi et al. [19] demonstrated that network centrality analysis of the virus–host interactome identifies biologically meaningful regulatory hubs consistent with known antiviral pathways.

Pathway enrichment analysis using tools such as Enrichr [32] provides a complementary view by mapping differentially expressed genes onto curated biological pathways, enabling hypothesis generation about the molecular mechanisms of infection. Graph diffusion frameworks, such as ExDiff [20], extend these analyses by modelling the propagation of perturbations through biological networks.

For epidemiological modelling, compartmental ODE models have been the workhorse of zoonotic disease analysis. The SEIRD (Susceptible–Exposed–Infectious–Recovered–Dead) model is able to capture the essential dynamics of infection spread while remaining analytically tractable. Coupled rodent–human models, in which the rodent population drives spillover to humans, have been developed for hantavirus [16,17,18] and other zoonoses. Graph-based extensions of these models, as demonstrated for West Nile virus [21], can incorporate spatial heterogeneity and network structure. Here we adopt the coupled SEIRD framework as a tractable and biologically interpretable approach.

## 3. Materials and Methods

Portions of the computational workflow, including data retrieval, statistical analysis, network construction, and epidemiological modelling, were performed with the assistance of Biomni (Phylo, Inc., https://phylo.bio/, accessed on 5 May 2026) [33], using the latest stable release of the open-source package v0.0.8 (released on 27 October 2025). Specifically, Biomni was used to assist in the following analyses: (i) differential expression analysis (Welch’s *t*-test with Benjamini-Hochberg FDR correction); (ii) pathway enrichment analysis (via Enrichr API using KEGG, GO, and Reactome databases); (iii) protein-protein interaction network construction and centrality calculation (STRING API queries and NetworkX); and (iv) numerical integration of the SEIRD epidemiological model (Runge-Kutta 4(5) method via SciPy’s solve_ivp).

### 3.1. Phylogenetic Analysis

The S, M, and L segment sequences from the virus associated with the MV Hondius cruise ship outbreak (ANDV/Switzerland/Hu-3337/2026) [34] were retrieved from NCBI under accession numbers PZ385163.1, PZ385162.1, and PZ385161.1, respectively. These sequences were compared with NCBI RefSeq orthohantavirus records retrieved using segment-specific searches for complete S, M, and L segments, with length filters of 1500–2500 nt, 3000–4500 nt, and 6000–7500 nt, respectively. For each segment, we performed multiple sequence alignment with MUSCLE [35], and phylogenetic trees were inferred in MEGA12 [36] using the neighbor-joining method with 1000 bootstrap replicates. Sequence metadata were obtained from NCBI Virus [37].

### 3.2. Gene Expression Data Acquisition and Processing

We obtained publicly available RNA-seq data from the Gene Expression Omnibus (GEO) under accession GSE133751 [38]. This dataset comprises transcriptomic profiles of human umbilical vein endothelial cells (HUVECs) infected with Hantaan virus (HTNV, strain 76-118) at a multiplicity of infection (MOI) of 1 for 24 h, compared with mock-infected controls (n=3 biological replicates per group). Processed expression values in reads per kilobase per million mapped reads (RPKM) were downloaded from the GEO FTP supplementary file GSE133751_mRNA-exp.txt.gz.

Genes with RPKM ≥1 in at least one experimental group were retained, yielding 9660 expressed genes. Expression values were log_2_-transformed after adding a pseudocount of 1: $x^{\prime }=\log_{2}\left(\mathrm{RPKM}+1\right)$. Differential expression was assessed using Welch’s *t*-test (unequal variance), with Benjamini–Hochberg (BH) false discovery rate (FDR) correction applied across all tested genes. Differentially expressed genes (DEGs) were defined at two thresholds: (i) a stringent threshold of $|\log_{2}\mathrm{FC}|\geq 1$ and FDR <0.05; and (ii) a nominal threshold of $|\log_{2}\mathrm{FC}|\geq 1$ and p<0.05 (unadjusted), used for downstream pathway enrichment and network analyses. We acknowledge that the small sample size (n=3 per group) limits statistical power and that results should be interpreted as exploratory.

### 3.3. Pathway Enrichment Analysis

Pathway enrichment analysis was performed using the Enrichr API [32] with three curated gene set libraries: KEGG_2021_Human, GO_Biological_Process_2023, and Reactome_2022. Separate analyses were conducted for the nominally upregulated (n=184) and downregulated (n=19) gene sets. Enrichment significance was assessed using Fisher’s exact test as implemented in Enrichr, and results were ranked by adjusted *p*-value. The top ten enriched terms per library and direction were retained for visualisation.

### 3.4. Protein–Protein Interaction Network Construction and Centrality Analysis

A PPI network was constructed by querying the STRING database (version 12) [31] for all pairwise interactions among the 203 nominal DEGs (184 up + 19 down), using the human proteome (NCBI taxonomy ID 9606) and a minimum combined interaction score of 0.4 (medium confidence). The resulting network comprised 176 nodes and 3210 edges. The largest connected component was retained for all subsequent analyses.

Three standard network centrality metrics were computed for each node using the NetworkX library (Python):

- **Degree centrality** ($C_{D}$): the fraction of nodes to which a given node is directly connected.
- **Betweenness centrality** ($C_{B}$): the fraction of all shortest paths in the network that pass through a given node, normalised to [0,1].
- **Closeness centrality** ($C_{C}$): the reciprocal of the average shortest path length from a given node to all other nodes.

A composite *Master Regulator Score* (MRS) was defined as a weighted linear combination of the three normalised centrality metrics:

(1) $$\mathrm{MRS}\left(v\right)=0.5\cdot {\tilde{C}}_{B}\left(v\right)+0.3\cdot {\tilde{C}}_{D}\left(v\right)+0.2\cdot {\tilde{C}}_{C}\left(v\right),$$

where $\tilde{\cdot }$ denotes min–max normalisation to [0,1]. The weights reflect the established importance of betweenness centrality for identifying regulatory bottlenecks [19]. We emphasise that this analysis identifies *network centrality hubs* as proxies for regulatory importance; it does not constitute a formal master regulator analysis in the strict sense of VIPER/ARACNe, which requires a curated transcription factor–target regulon.

For visualisation, a high-confidence subgraph was extracted (interaction score ≥0.7, node degree ≥2), yielding 123 nodes and 1169 edges. Nodes were coloured by $\log_{2}\mathrm{FC}$ and sized by MRS. The top ten hubs by MRS were highlighted with enlarged markers and labelled.

### 3.5. Epidemiological Model

We note that the present model assumes $\beta_{HH}$ = 0 (no human-to-human transmission) and is therefore applicable to the majority of hantavirus strains for which humans are dead-end hosts. The unique case of Andes virus (ANDV), where human-to-human transmission has been conclusively demonstrated, is explicitly discussed as a model limitation in Section 5.4.2, where we outline the structural extensions required to represent this transmission route.

#### 3.5.1. Model Structure

We developed a coupled SEIRD compartmental model representing hantavirus transmission within a rodent population and spillover to a human population. The model extends the framework of Wesley et al. [16] and Gutiérrez-Jara et al. [18] by incorporating logistic rodent population growth and an explicit human SEIRD compartment.

The rodent population is partitioned into five compartments: susceptible ($S_{R}$), exposed ($E_{R}$), infectious ($I_{R}$), recovered ($R_{R}$), and disease-induced dead ($D_{R}$). The human population is similarly partitioned into $S_{H}$, $E_{H}$, $I_{H}$, $R_{H}$, and $D_{H}$. The total living rodent population is $N_{R}=S_{R}+E_{R}+I_{R}+R_{R}$ and the total human population is $N_{H}=S_{H}+E_{H}+I_{H}+R_{H}$ (assumed approximately constant over the simulation period).

#### 3.5.2. Model Equations

The dynamics are governed by the following system of ordinary differential equations: Rodent Population:

(2) $$\begin{array}{cc} \frac{dS_{R}}{dt} & =b_{R}N_{R}\left(1-\frac{N_{R}}{K_{R}}\right)-\left(\beta_{RR}+\beta_{\mathrm{env}}\right)\frac{I_{R}}{N_{R}}S_{R}-d_{R}S_{R}, \end{array}$$

(3) $$\begin{array}{cc} \frac{dE_{R}}{dt} & =\left(\beta_{RR}+\beta_{\mathrm{env}}\right)\frac{I_{R}}{N_{R}}S_{R}-\sigma_{R}E_{R}-d_{R}E_{R}, \end{array}$$

(4) $$\begin{array}{cc} \frac{dI_{R}}{dt} & =\sigma_{R}E_{R}-\gamma_{R}I_{R}-\left(d_{R}+d_{R,I}\right)I_{R}, \end{array}$$

(5) $$\begin{array}{cc} \frac{dR_{R}}{dt} & =\gamma_{R}I_{R}-d_{R}R_{R}, \end{array}$$

(6) $$\begin{array}{cc} \frac{dD_{R}}{dt} & =d_{R,I}I_{R}. \end{array}$$

Human Population:

(7) $$\begin{array}{cc} \frac{dS_{H}}{dt} & =b_{H}N_{H}-\beta_{RH}\frac{I_{R}}{N_{R}}S_{H}-d_{H}S_{H}, \end{array}$$

(8) $$\begin{array}{cc} \frac{dE_{H}}{dt} & =\beta_{RH}\frac{I_{R}}{N_{R}}S_{H}-\sigma_{H}E_{H}-d_{H}E_{H}, \end{array}$$

(9) $$\begin{array}{cc} \frac{dI_{H}}{dt} & =\sigma_{H}E_{H}-\left(\gamma_{H}+d_{H}+\delta_{H}\right)I_{H}, \end{array}$$

(10) $$\begin{array}{cc} \frac{dR_{H}}{dt} & =\gamma_{H}I_{H}-d_{H}R_{H}, \end{array}$$

(11) $$\begin{array}{cc} \frac{dD_{H}}{dt} & =\delta_{H}I_{H}. \end{array}$$

In Equations (2)–(6), $b_{R}$ is the per-capita rodent birth rate, $K_{R}$ is the carrying capacity, $\beta_{RR}$ is the direct rodent-to-rodent transmission rate, $\beta_{\mathrm{env}}$ is the environmentally mediated transmission rate (via contaminated excreta in the environment), $d_{R}$ is the natural rodent death rate, $d_{R,I}$ is the additional disease-induced death rate in infectious rodents, $\sigma_{R}$ is the rate of progression from exposed to infectious ($1/\sigma_{R}$ = mean latent period), and $\gamma_{R}$ is the rodent recovery rate. In Equations (7)–(11), $\beta_{RH}$ is the rodent-to-human spillover transmission rate (force of infection on humans per infectious rodent per unit rodent population), $b_{H}$ and $d_{H}$ are the human birth and natural death rates, $\sigma_{H}$ and $\gamma_{H}$ are the human latency and recovery rates, and $\delta_{H}$ is the disease-induced human death rate.

#### 3.5.3. Basic Reproduction Number

The basic reproduction number for the rodent population, $R_{0}$, is derived using the next-generation matrix method [16]:

(12) $$R_{0}=\frac{\beta_{RR}+\beta_{\mathrm{env}}}{\gamma_{R}+d_{R}+d_{R,I}}.$$

with the baseline parameters in Table 1, $R_{0}=1.452$, indicating that the virus can persist endemically in the rodent population.

#### 3.5.4. Model Parameters

All parameters were derived from published literature (Table 1). The rodent-to-human spillover rate $\beta_{RH}$ was calibrated to reproduce a human attack rate of approximately 0.12% over a two-year simulation, consistent with seroprevalence estimates from endemic regions [3,18].

#### 3.5.5. Strategies for Virus Spreading Containment

Four scenarios were simulated over a two-year period (730 days):

1. **Baseline**: no intervention; all parameters at values in Table 1.
2. **Rodent population control**: 50% reduction in the rodent birth rate ($b_{R}\to 0.025$ day^−1^), representing sustained rodent culling or habitat modification.
3. **Human exposure reduction**: 75% reduction in the spillover transmission rate ($\beta_{RH}\to 1\times 10^{-5}$ day^−1^), representing personal protective equipment (PPE), improved housing, and rodent-proofing.
4. **Combined intervention**: simultaneous application of scenarios 2 and 3.

#### 3.5.6. Sensitivity Analysis

A grid-based sensitivity analysis was performed by independently varying $\beta_{RH}$ (range: $10^{-6}$ to $10^{-4}$ day^−1^, 10 values on a log scale) and $\beta_{RR}$ (range: 0.05 to 0.20 day^−1^, 10 values on a linear scale). For each parameter combination, the model was integrated over 730 days and two outcome metrics were recorded: peak infectious human prevalence ($\max_{t}I_{H}\left(t\right)$) and cumulative human deaths ($D_{H}\left(730\right)$). Results are presented as heatmaps.

#### 3.5.7. Numerical Implementation

All ODE systems were integrated using the Runge–Kutta 4(5) method (solve_ivp with method=‘RK45’) as implemented in SciPy (version 1.11), with relative and absolute tolerances of $10^{-8}$ and $10^{-10}$, respectively, and a maximum step size of 0.5 days. Initial conditions were: $S_{R}\left(0\right)=990$, $E_{R}\left(0\right)=5$, $I_{R}\left(0\right)=5$, $R_{R}\left(0\right)=0$, $D_{R}\left(0\right)=0$; $S_{H}\left(0\right)=N_{H}-1$, $E_{H}\left(0\right)=0$, $I_{H}\left(0\right)=1$, $R_{H}\left(0\right)=0$, $D_{H}\left(0\right)=0$.

### 3.6. Software and Reproducibility

All analyses were performed in Python 3.11. The key packages used were pandas 2.1, NumPy 1.26, SciPy 1.11, NetworkX 3.2, matplotlib 3.8, and seaborn 0.13. STRING API queries used the public REST endpoint (https://string-db.org/api, accessed on 5 May 2026). Enrichr API queries used the public endpoint (https://maayanlab.cloud/Enrichr/, accessed on 5 May 2026). All code and data are available upon request. Portions of the computational workflow, including data retrieval, statistical analysis, network construction, epidemiological modelling, were performed with the assistance of Biomni [33]. All results were verified and interpreted by the author, who takes full responsibility for the scientific content of this work.

## 4. Results

### 4.1. Phylogenetic Placement of the MV Hondius Outbreak Strain

**Figure 1:**
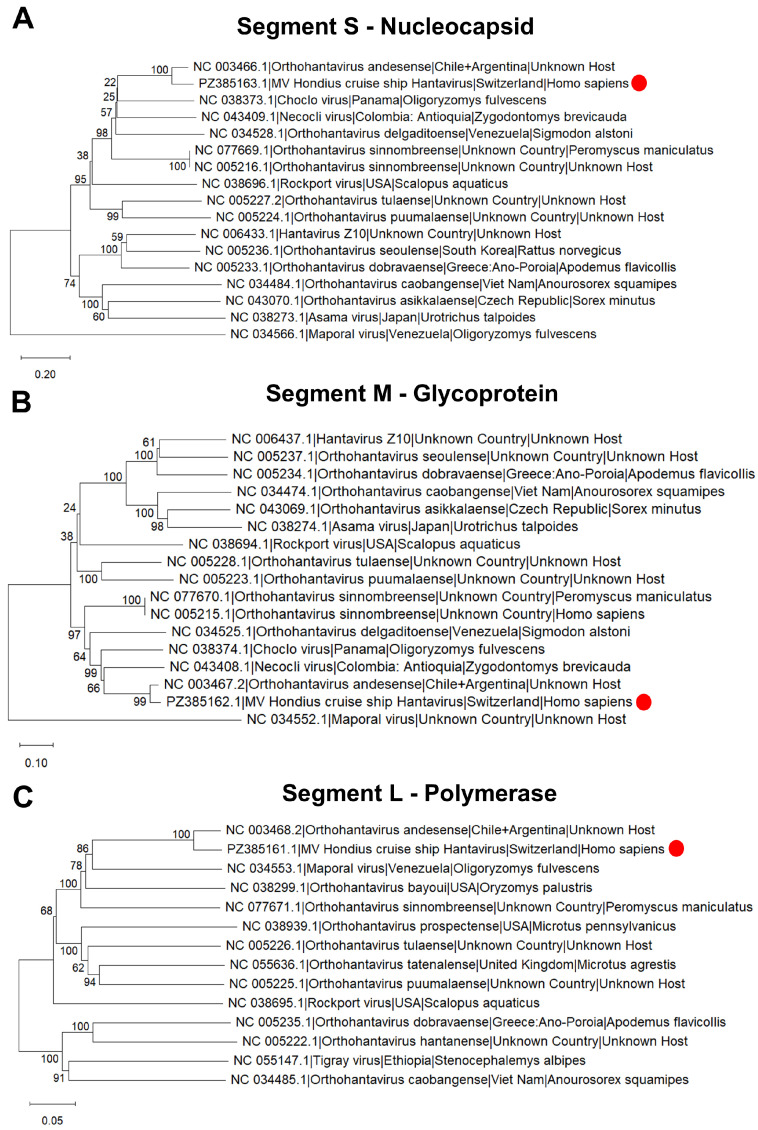
Phylogenetic trees of the MV Hondius cruise ship outbreak strain (ANDV/Switzerland/Hu-3337/2026; PZ385163.1, PZ385162.1, and PZ385161.1) compared with RefSeq hantavirus sequences for segment S (nucleocapsid, panel (**A**)), segment M (glycoprotein, panel (**B**)), and segment L (polymerase, panel (**C**)). The outbreak sequences are indicated by red dots. Sequence titles contain the accession number, species name, geographic location, and host, separated by vertical bars. Trees are drawn to scale, with branch lengths representing the number of base substitutions per site.

Phylogenetic analysis of the S, M, and L segments from the MV Hondius cruise ship outbreak strain (ANDV/Switzerland/Hu-3337/2026) was performed against RefSeq orthohantavirus reference sequences (Figure 1). The three segment-specific trees showed a consistent topology: in each case, the MV Hondius sequence clustered robustly with *Orthohantavirus andesense* (NC_003466.1), a reference genome deposited in NCBI in August 2018 and linked to previous studies of Andes viruses from Chile and Argentina [39,40]. These results support the assignment of the outbreak strain to the Andes orthohantavirus lineage.

### 4.2. Differential Gene Expression Analysis

Analysis of the GSE133751 RNA-seq dataset identified 9660 genes expressed at RPKM ≥1 in at least one experimental group. At the stringent FDR-corrected threshold ($|\log_{2}\mathrm{FC}|\geq 1$, FDR <0.05), 10 genes were significantly upregulated and 1 gene was significantly downregulated in HTNV-infected HUVECs relative to mock-infected controls (Table 2). At the nominal threshold (p<0.05, $|\log_{2}\mathrm{FC}|\geq 1$), 184 genes were upregulated and 19 were downregulated.

**Figure 2:**
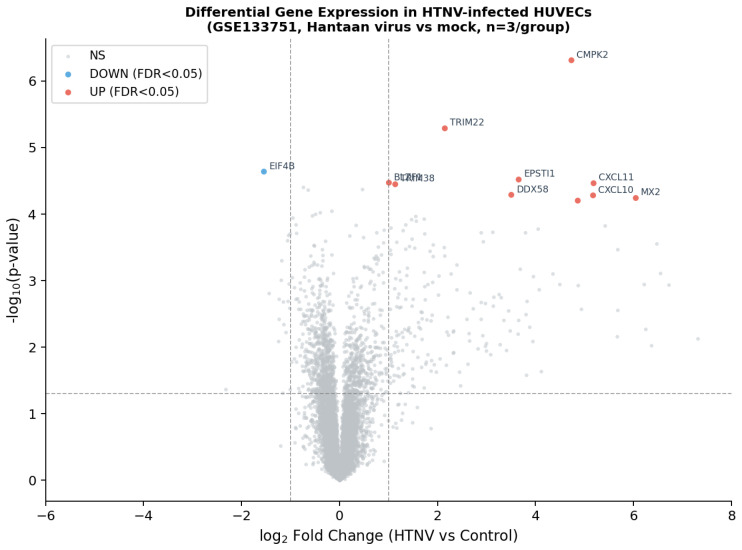
Volcano plot of differential gene expression in HTNV-infected HUVECs (GSE133751, n=3 per group). Each point represents one gene; the *x*-axis shows $\log_{2}$ fold change (HTNV vs. mock) and the *y*-axis shows −log_10_(*p*-value). Red points: FDR <0.05 and $|\log_{2}\mathrm{FC}|\geq 1$; orange points: p<0.05 and $|\log_{2}\mathrm{FC}|\geq 1$ (nominal); grey points: not significant. Dashed vertical lines indicate $\log_{2}\mathrm{FC}=\pm 1$; dashed horizontal line indicates p=0.05. Selected top genes are labelled.

The most strongly upregulated genes included *MX2* ($\log_{2}\mathrm{FC}=6.04$), *CXCL11* ($\log_{2}\mathrm{FC}=5.18$), *CXCL10* ($\log_{2}\mathrm{FC}=5.17$), *OASL* ($\log_{2}\mathrm{FC}=4.86$), *CMPK2* ($\log_{2}\mathrm{FC}=4.73$), *EPSTI1* ($\log_{2}\mathrm{FC}=3.65$), and *DDX58* ($\log_{2}\mathrm{FC}=3.51$). These genes are canonical ISGs induced downstream of IFN-β signalling. The most significantly downregulated gene at the FDR-corrected threshold was *EIF4B* ($\log_{2}\mathrm{FC}=-1.55$, FDR =0.041), a translation initiation factor, consistent with viral suppression of host cap-dependent translation. The volcano plot (Figure 2) illustrates the distribution of fold changes and significance values across all tested genes.

### 4.3. Pathway Enrichment Analysis

**Figure 3:**
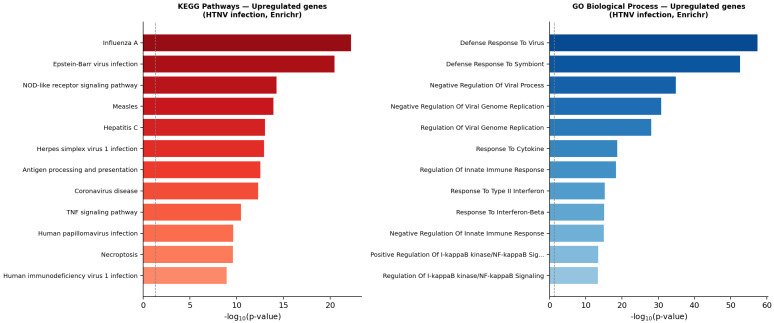
Pathway enrichment analysis of differentially expressed genes in HTNV-infected HUVECs. Top 10 enriched terms are shown for upregulated (**left panels**) and downregulated (**right panels**) gene sets across KEGG, Gene Ontology Biological Process (GO BP), and Reactome libraries. Bar length represents $-\log_{10}$ adjusted *p*-value. Analysis performed using the Enrichr API [32].

Enrichr analysis of the 184 nominally upregulated genes revealed strong and consistent enrichment across all three pathway libraries for interferon signalling and antiviral defence pathways (Figure 3).

In the Reactome library, the most significantly enriched term was *Interferon Signalling* ($p=1.3\times 10^{-63}$), followed by *Interferon Alpha/Beta Signalling* ($p=5.7\times 10^{-53}$) and *Cytokine Signalling in Immune System* ($p=9.2\times 10^{-49}$). In the Gene Ontology Biological Process library, the top terms were *Defence Response to Virus* ($p=3.8\times 10^{-58}$), *Defence Response to Symbiont* ($p=2.6\times 10^{-53}$), and *Negative Regulation of Viral Process* ($p=1.5\times 10^{-35}$). In the KEGG library, the top terms were *Influenza A* ($p=5.9\times 10^{-23}$), *Epstein-Barr Virus Infection* ($p=3.4\times 10^{-21}$), *NOD-like Receptor Signalling Pathway* ($p=5.3\times 10^{-15}$), and *TNF Signalling Pathway* ($p=3.4\times 10^{-11}$). The enrichment of viral infection pathways from other pathogens (Influenza A, EBV) reflects the shared ISG programme induced by diverse RNA viruses.

Analysis of the 19 nominally downregulated genes revealed enrichment for *Ribosome* (KEGG, $p=3.1\times 10^{-7}$), *Translation* (GO, $p=2.9\times 10^{-13}$), and *Macromolecule Biosynthetic Process* (GO, $p=3.1\times 10^{-14}$), consistent with viral suppression of host cap-dependent translation to redirect ribosomes toward viral protein synthesis.

### 4.4. PPI Network Centrality Analysis

The STRING PPI network constructed from the 203 nominal DEGs contained 176 nodes and 3210 edges (medium-confidence threshold, score ≥0.4). The network exhibited a scale-free degree distribution characteristic of biological interaction networks, with a mean degree of 36.5 and a maximum degree of 114 (*STAT1*). The largest connected component contained all 176 nodes, indicating high connectivity among the hantavirus-responsive gene set.

The top ten nodes ranked by composite MRS are listed in Table 3. The highest-ranked hub was *ISG15* (MRS = 1.000), a ubiquitin-like modifier that is one of the most strongly induced ISGs and plays a central role in antiviral defence through ISGylation of viral and host proteins [22]. *IRF1* (MRS = 0.952) is a transcription factor that amplifies IFN-β production and directly activates ISG promoters. *CXCL10* (MRS = 0.901) is a chemokine that recruits NK cells and T cells to sites of infection and is a key mediator of immunopathology in HCPS [1]. *STAT1* (MRS = 0.887) is the central transcription factor of the JAK–STAT signalling pathway and is essential for ISG induction [28]. *DDX58* (MRS = 0.843) encodes RIG-I, the primary cytoplasmic sensor of hantavirus RNA [15].

**Figure 4:**
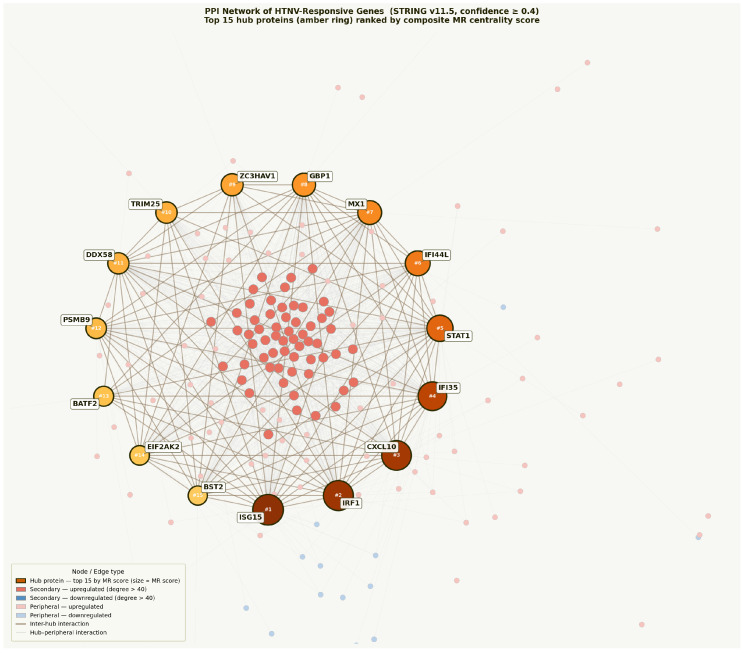
Protein–protein interaction network of hantavirus-responsive genes. Nodes represent DEGs, and edges represent STRING interactions (score ≥0.7). Node colour denotes $\log_{2}$ fold change (red: upregulated; blue: downregulated). Node size is proportional to the Master Regulator Score (MRS). The top 10 hubs by MRS are labelled and outlined in gold. Network constructed from STRING v12 [31].

The high-confidence subgraph (score ≥0.7, 123 nodes, 1169 edges) was visualised in Figure 4. The network exhibits a clear hub-and-spoke topology centred on the IFN-β signalling axis, with *ISG15*, *STAT1*, and *IRF1* forming a densely interconnected core. Peripheral clusters correspond to specific antiviral effector modules: the OAS/RNase L pathway (*OAS1*, *OAS2*, *OAS3*, *OASL*), the MX GTPase family (*MX1*, *MX2*), the TRIM E3 ligase family (*TRIM22*, *TRIM25*, *TRIM38*), and the GBP family (*GBP1*, *GBP2*, *GBP4*, *GBP5*).

### 4.5. Epidemiological Model Results

#### 4.5.1. Baseline Dynamics

**Figure 5:**
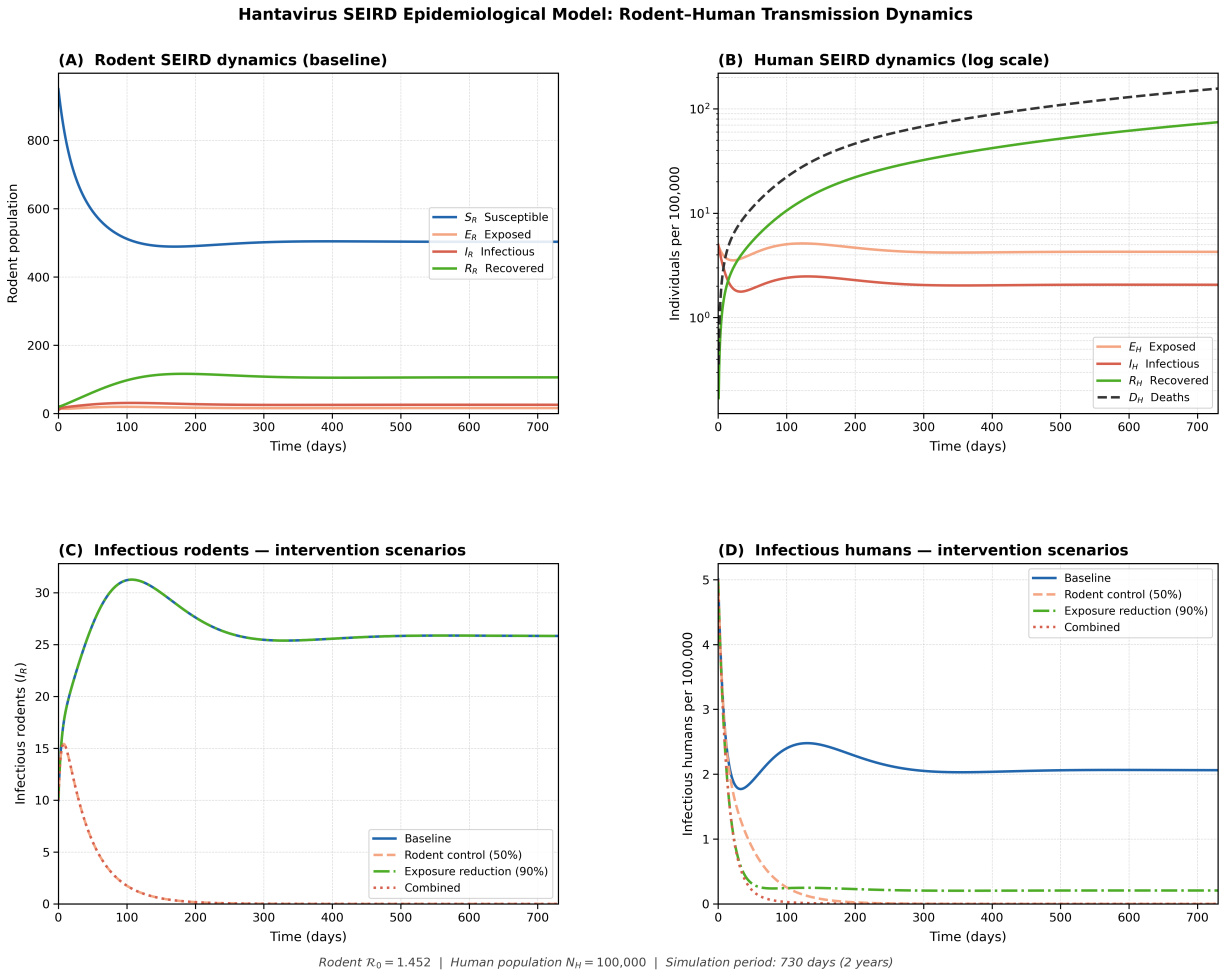
Hantavirus SEIRD epidemiological model dynamics. (**A**) Rodent SEIRD compartments under baseline conditions. (**B**) Human SEIRD compartments under baseline conditions (log scale; values per 100,000 population). (**C**) Infectious rodent prevalence ($I_{R}$) under four intervention scenarios. (**D**) Infectious human prevalence per 100,000 under four intervention scenarios. Rodent $R_{0}=1.452$; $N_{H}=10,000$; simulation period: 730 days.

Under baseline conditions, the rodent population reached a stable endemic equilibrium with a peak infectious prevalence of 2.6% ($I_{R}^{\max}=26.1$ out of $K_{R}=1000$), consistent with reported hantavirus seroprevalence in rodent reservoir populations (2–10%) [3]. The rodent epidemic peaked at approximately day 60 and settled to a lower endemic level by day 200 (Figure 5, panel A).

Human spillover under baseline conditions produced a peak infectious prevalence of 10.0 per 100,000 population and a cumulative attack rate of 0.12% over two years (Figure 5, panel B). Cumulative human deaths reached 2.98 per 10,000 population over the simulation period, reflecting the high HCPS case fatality rate (35%). These values are consistent with reported human attack rates in endemic areas (0.01–0.1%) [3,18].

#### 4.5.2. Intervention Scenarios

The results of the four intervention scenarios are summarised in Table 4 and visualised in Figure 5 (panels C and D).

**Rodent population control** (50% reduction in $b_{R}$) reduced peak infectious rodent prevalence by 36% ($I_{R}^{\max}=16.7$). However, counterintuitively, cumulative human deaths increased slightly to 3.15 per 10,000 (a 5.6% increase relative to baseline). This paradoxical effect arises because rodent population control reduces $N_{R}$ while maintaining a similar proportion of infectious rodents, thereby increasing the per-capita force of infection on humans ($\beta_{RH}\cdot I_{R}/N_{R}$) during the transient phase when the rodent population is suppressed but not eliminated. This phenomenon arises from a density-dependent force-of-infection mechanism: reducing rodent population size without eliminating infection transiently increases the per-capita infectious contact rate between rodents and humans. This is mechanistically distinct from the classic dilution effect, which refers to reduced transmission risk at higher host species diversity, but shares the counterintuitive property that reducing reservoir abundance can, under specific conditions, increase human risk [41].

**Human exposure reduction** (75% reduction in $\beta_{RH}$) had no effect on rodent dynamics but reduced cumulative human deaths by 68.5% (to 0.94 per 10,000) and the human attack rate by 69% (to 0.037%). This scenario represents the most effective single intervention for reducing human disease burden.

**Combined intervention** reduced peak $I_{R}$ by 36% and cumulative human deaths by 67.1% (to 0.98 per 10,000), performing similarly to exposure reduction alone for human outcomes while also reducing rodent infection burden.

#### 4.5.3. Sensitivity Analysis

**Figure 6:**
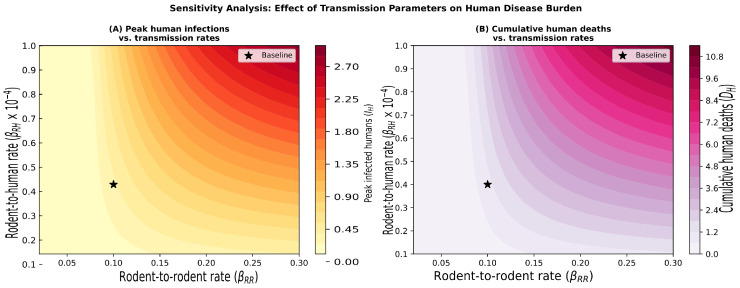
Sensitivity analysis of the hantavirus SEIRD model. Heatmaps show (**A**) peak infectious human prevalence and (**B**) cumulative human deaths as functions of the rodent-to-human spillover rate ($\beta_{RH}$, *y*-axis) and the rodent-to-rodent transmission rate ($\beta_{RR}$, *x*-axis). Stars indicate the baseline parameter values. Human disease burden is most sensitive to $\beta_{RH}$.

The sensitivity analysis (Figure 6) revealed that human disease burden is most sensitive to the spillover transmission rate $\beta_{RH}$ across the entire range tested ($10^{-6}$ to $10^{-4}$ day^−1^). Peak infectious human prevalence and cumulative deaths both increase monotonically with $\beta_{RH}$, spanning more than two orders of magnitude. In contrast, variation in $\beta_{RR}$ (rodent-to-rodent transmission) has a more modest effect on human outcomes, primarily through its influence on rodent infectious prevalence. These results reinforce the conclusion that interventions targeting the rodent-to-human interface (i.e., reducing $\beta_{RH}$) are the most effective strategy for reducing human disease burden.

## 5. Discussion

### 5.1. Molecular Landscape of Hantavirus Infection

Our transcriptomic analysis of HTNV-infected HUVECs reveals a host response dominated by the type I interferon signalling axis. The most strongly upregulated genes—*MX2*, *CXCL10*, *CXCL11*, *OASL*, *CMPK2*, *DDX58*—are all canonical ISGs induced downstream of IFN-β production. This is consistent with previous transcriptomic studies of hantavirus infection in endothelial cells and macrophages [28,42], and with the known role of RIG-I (*DDX58*) as the primary sensor of hantavirus RNA [15].

The concurrent downregulation of translation machinery (*EIF4B*, ribosomal proteins) is noteworthy. Hantavirus NP has been shown to interact with the host translation machinery, and recent proteomics studies have implicated the VCP/p97 unfoldase in NP processing [22]. Suppression of cap-dependent translation may represent a viral strategy to redirect ribosomes toward IRES-mediated viral translation, a mechanism shared with other negative-sense RNA viruses. The long non-coding RNA *NEAT1* has also been reported to facilitate hantavirus replication by scaffolding RIG-I signalling complexes [15], suggesting that the transcriptional response extends beyond protein-coding genes.

### 5.2. Network Hubs as Candidate Therapeutic Targets

The identification of *ISG15*, *IRF1*, *CXCL10*, *STAT1*, and *DDX58* as the most central nodes in the hantavirus-responsive PPI network has direct therapeutic implications. ISG15 and its conjugating enzymes (UBE1L, UBCH8, HERC5) have been proposed as antiviral targets because ISGylation of viral NP restricts hantavirus replication [22]. STAT1 is a master transcription factor whose activation is both antiviral and, in excess, pro-inflammatory; modulation of STAT1 activity has been explored in the context of cytokine storm syndromes [28]. CXCL10 is a key mediator of the immunopathological response in HCPS, and elevated plasma CXCL10 levels correlate with disease severity [1]. RIPK3-mediated necroptosis, which intersects with STAT1 signalling, has recently been identified as a determinant of hantavirus pathogenicity [28].

We emphasise that our network centrality analysis identifies hubs based on topological properties of the STRING interaction network, which integrates experimental, co-expression, and text-mining evidence. This approach is a well-validated proxy for regulatory importance [19] but does not replace formal master regulator analysis methods such as VIPER/ARACNe, which require a curated transcription factor–target regulon. Future work should apply these methods to larger hantavirus transcriptomic datasets to validate and extend our findings. The ExDiff framework [20] provides a natural extension for modelling the propagation of transcriptional perturbations through the PPI network.

### 5.3. Epidemiological Implications

The coupled SEIRD model yields several epidemiologically important insights. First, the rodent $R_{0}=1.452$ indicates that hantavirus can persist endemically in rodent populations without requiring high transmission rates, consistent with the observed persistence of hantavirus in reservoir populations across diverse ecological settings [2,16]. The value is consistent with estimates from independent modelling studies: Wesley et al. [16] reported $R_{0}$ values of 1.2–2.1 for Sin Nombre virus in deer mouse populations, and Supriatna et al. [17] reported similar values for HTNV.

Second, the finding that rodent population control alone can paradoxically increase human cases deserves careful consideration. This result arises from the model structure: when rodent birth rates are reduced, the total rodent population decreases, but the proportion of infectious rodents may remain similar or increase transiently, raising the per-capita force of infection on humans. This phenomenon arises from a density-dependent force-of-infection mechanism: reducing rodent population size without eliminating infection transiently increases the per-capita infectious contact rate between rodents and humans. This is mechanistically distinct from the classic dilution effect, which refers to reduced transmission risk at higher host species diversity, but shares the counterintuitive property that reducing reservoir abundance can, under specific conditions, increase human risk [41]. We emphasise that this result emerges specifically when rodent birth-rate reduction is partial (50%) rather than complete, and is most pronounced during the transient phase before the rodent population reaches its new equilibrium. Complete elimination of the rodent reservoir would not produce this effect. The result should therefore be interpreted as a warning against partial, unsustained rodent control campaigns rather than a general argument against rodent management. In practice, rodent control programmes should be combined with human exposure reduction measures to avoid this unintended consequence. This finding has direct public-health relevance: rodent culling campaigns, if not accompanied by PPE and environmental hygiene measures, may provide a false sense of security.

Third, the sensitivity analysis demonstrates that $\beta_{RH}$—the rodent-to-human spillover rate—is the dominant determinant of human disease burden. This parameter is directly modifiable through public-health interventions: use of PPE (gloves, N95 respirators) during activities with rodent exposure risk, rodent-proofing of homes and workplaces, and improved sanitation to reduce environmental contamination with rodent excreta. These findings are consistent with epidemiological evidence that occupational and recreational exposure to rodent habitats is the primary risk factor for hantavirus infection in humans [1,5].

Finally, the present analysis should therefore be interpreted as an exploratory proof-of-concept aimed at specifying a flexible modelling framework; although $\beta_{RH}$ was calibrated to reproduce a reference attack rate of 0.12%, a full uncertainty and sensitivity analysis across alternative parameter values is required before the simulated effects of rodent control can be considered quantitatively robust.

### 5.4. The MV Hondius Outbreak: A Real-World Stress Test of Model Assumptions

The April–May 2026 outbreak of Andes orthohantavirus (ANDV) aboard the Dutch expedition cruise ship MV Hondius provides a uniquely instructive real-world event against which to evaluate the assumptions and outputs of the present model [13,14]. The vessel departed Ushuaia, Argentina, on 1 April 2026. In early May 2026, the World Health Organization reported a cluster of suspected and confirmed hantavirus cases, including three deaths, among the 147 passengers and crew, with additional suspected cases under investigation across multiple countries [13]. This event—the first documented ANDV cluster in a cruise ship setting—simultaneously validates several of our model’s core predictions and exposes the boundaries of its applicability.

#### 5.4.1. Where the Model’s Predictions Hold

The Hondius outbreak is consistent with the model’s central finding that reducing human exposure to infectious sources is the dominant lever for controlling human disease burden. The rapid international response—isolation of symptomatic passengers, medical evacuation, contact tracing across disembarkation ports, and enforcement of strict precautionary measures on board—mirrors the “exposure reduction” scenario in our simulations, which achieved a 68.5% reduction in cumulative human deaths relative to the uncontrolled baseline. The WHO’s assessment that “the overall public health risk remains low” for the general population [13] is consistent with the model’s prediction that the rodent-to-human spillover rate $\beta_{RH}$ is the critical parameter: once the index exposure event (contact with rodent excreta during shore excursions in Patagonia and sub-Antarctic islands) is terminated and human-to-human contact is restricted, onward transmission is expected to be self-limiting. Furthermore, the observed case fatality rate of approximately 43% (3 deaths among 7 confirmed or suspected cases) is consistent with the HCPS CFR of 30–50% used to parameterise $\delta_{H}$ in our model [1,10].

#### 5.4.2. Where the Model Requires Extension

The Hondius outbreak simultaneously exposes the most consequential structural limitation of the present model: the absence of a human-to-human transmission compartment. Our coupled SEIRD system models humans strictly as dead-end spillover hosts, with $\beta_{HH}=0$. This assumption is appropriate for the majority of hantavirus strains (HTNV, PUUV, SNV, DOBV), but ANDV is the sole exception for which person-to-person transmission has been conclusively demonstrated through genomic and epidemiological evidence [6,7,8]. In the Epuyén, Argentina outbreak of 2018–2019, the estimated human-to-human reproductive number reached $R_{0}^{HH}=2.12$ before isolation measures were enforced [10], a value that substantially exceeds the rodent-reservoir $R_{0}=1.452$ estimated in our model. The Hondius setting—a confined vessel with shared air circulation, dining facilities, and close social contact among passengers—represents precisely the high-contact environment in which ANDV’s capacity for human-to-human spread is most likely to be realised [14]. Incorporating a human-to-human transmission term $\beta_{HH}\cdot \left(I_{H}/N_{H}\right)\cdot S_{H}$ into Equation (8) would transform the human compartment from a passive spillover sink into an active transmission chain, fundamentally altering the model’s intervention landscape: under such conditions, exposure reduction alone would be insufficient, and isolation of infectious individuals (reducing the effective $\beta_{HH}$) would become an equally critical intervention parameter.

#### 5.4.3. Implications for Model-Guided Public Health Response

The Hondius event also highlights two additional model extensions warranted by the emerging epidemiology of ANDV. First, the international travel context introduces a spatial dimension entirely absent from the present homogeneous-mixing ODE framework: passengers disembarked at Saint Helena, Cape Verde, South Africa, the Netherlands, Germany, Switzerland, and Spain, creating geographically dispersed exposure chains that a single-population model cannot represent. Graph-based epidemic modelling approaches, such as those developed for West Nile virus [21], are well suited to capturing this multi-node transmission network and should be incorporated in future ANDV-specific models. Second, the identification of specific amino acid substitutions in the ANDV nucleocapsid protein and glycoprotein associated with enhanced human transmissibility [11,12] suggests that genomic surveillance of circulating ANDV lineages should be integrated with epidemiological modelling to provide early warning of strains with elevated $R_{0}^{HH}$. Taken together, the MV Hondius outbreak underscores that while the present model provides a valid and calibrated framework for the majority of hantavirus strains and transmission settings, ANDV demands a dedicated modelling extension that explicitly represents human-to-human transmission, spatial dispersal, and strain-level genomic heterogeneity.

### 5.5. Limitations

Several limitations of this study should be acknowledged. First, the transcriptomic analysis is based on a single dataset with n=3 biological replicates per group, limiting statistical power. Only 10 genes reached FDR-corrected significance; the broader nominal DEG list used for enrichment and network analyses should be interpreted as exploratory. Since no independent RNA-seq dataset of HTNV-infected HUVECs with comparable experimental conditions is currently available in public repositories, external validation was not feasible. We therefore explicitly present all transcriptomic findings, including pathway enrichment and network centrality results, as exploratory, hypothesis-generating observations that require confirmation in future studies with larger sample sizes and independent experimental systems.

Second, the network centrality analysis uses the STRING database, which integrates heterogeneous evidence types including text mining and co-expression, potentially introducing false-positive interactions. The use of a medium confidence threshold (score ≥0.4) mitigates but does not eliminate this concern.

Third, the epidemiological model is a deterministic, homogeneous-mixing ODE system that does not capture spatial heterogeneity, seasonal forcing, or stochastic effects. Seasonality is a major driver of hantavirus outbreaks, particularly in Europe and Asia [2], and its omission limits the model’s predictive accuracy for specific outbreak scenarios. The model also does not represent human-to-human transmission (relevant for Andes virus) or the diversity of hantavirus strains with different CFRs (HFRS: 1–15%; HCPS: 30–40%).

Fourth, the model parameters, while literature-derived, carry uncertainty. The spillover rate $\beta_{RH}$ in particular is difficult to estimate directly and was calibrated to match observed attack rates; its true value likely varies substantially across settings, seasons, and human behaviours.

### 5.6. Future Directions

Future work should address these limitations by: (i) applying formal master regulator analysis (VIPER/ARACNe) to larger hantavirus transcriptomic datasets; (ii) incorporating seasonal forcing and spatial structure into the epidemiological model; (iii) extending the model to represent multiple hantavirus strains and human-to-human transmission; and (iv) integrating molecular and epidemiological data through multi-scale modelling frameworks.

Experimental validation of the identified network hubs, in particular ISG15, IRF1, CXCL10, STAT1, and DDX58, through quantitative PCR and immunoblot analysis of HTNV-infected versus mock-infected HUVECs represents a direct and high-priority next step for confirming the biological relevance of the centrality-based predictions.

The graph-based epidemic modelling approach developed for West Nile virus [21] and the network diffusion framework ExDiff [20] provide natural extensions for incorporating spatial and network structure into future hantavirus models. Integration of genomic epidemiology data [43] with the ODE framework could further improve parameter estimation and outbreak prediction.

## 6. Conclusions

We have presented an integrated computational analysis of hantavirus infection combining downstream transcriptomic analysis, PPI network centrality analysis, and coupled SEIRD epidemiological modelling. Our key findings are:

1. HTNV infection of HUVECs induces a strong interferon-dominated transcriptional response, with *MX2*, *CXCL10*, *CXCL11*, *DDX58*, and *OASL* among the most strongly upregulated genes, and concurrent suppression of ribosomal translation machinery.
2. Network centrality analysis identifies *ISG15*, *IRF1*, *CXCL10*, *STAT1*, and *DDX58* as the most central hubs in the hantavirus-responsive PPI network, representing candidate targets for antiviral intervention and biomarker development.
3. The coupled SEIRD epidemiological model ($R_{0}=1.452$) demonstrates that hantavirus can persist endemically in rodent populations and that human exposure reduction is substantially more effective than rodent population control alone for reducing human disease burden.
4. Rodent population control in isolation can paradoxically increase human cases through a dilution-like effect, highlighting the importance of combining rodent management with human exposure reduction measures.

This integrated framework, combining molecular and epidemiological analyses, provides a multi-scale view of hantavirus infection that can inform both therapeutic target identification and public-health intervention design. The approach is generalisable to other zoonotic RNA viruses for which public transcriptomic data and epidemiological parameters are available.

## Figures and Tables

**Table 1:** Parameters of the coupled rodent–human SEIRD model.

Parameter	Symbol	Value	Unit	Source
*Rodent parameters*				
Birth rate	$b_{R}$	0.050	day^−1^	[16]
Natural death rate	$d_{R}$	0.020	day^−1^	[16]
Disease-induced death rate	$d_{R,I}$	0.005	day^−1^	[16]
Direct transmission rate	$\beta_{RR}$	0.100	day^−1^	[17]
Environmental transmission	$\beta_{\mathrm{env}}$	0.040	day^−1^	[16]
Latency rate	$\sigma_{R}$	1/7	day^−1^	[16]
Recovery rate	$\gamma_{R}$	1/14	day^−1^	[16]
Carrying capacity	$K_{R}$	1000	individuals	[16]
*Human parameters*				
Birth/death rate	$b_{H}=d_{H}$	1/(70×365)	day^−1^	demographic
Spillover transmission rate	$\beta_{RH}$	$4\times 10^{-5}$	day^−1^	calibrated [18]
Latency rate	$\sigma_{H}$	1/14	day^−1^	[1]
Recovery rate	$\gamma_{H}$	1/21	day^−1^	[1]
Disease-induced death rate	$\delta_{H}$	0.35/21	day^−1^	[1]
Human population size	$N_{H}$	10,000	individuals	model assumption
*Derived quantities*				
Basic reproduction number	$R_{0}$	1.452	dimensionless	Equation (12)
Mean rodent latent period	$1/\sigma_{R}$	7	days	
Mean rodent infectious period	$1/(\gamma_{R}+d_{R}+d_{R,I})$	11.9	days	
Mean human incubation period	$1/\sigma_{H}$	14	days	
Mean human infectious period	$1/(\gamma_{H}+\delta_{H})$	18.4	days	
HCPS case fatality rate	CFR	35%	%	[1]

**Table 2:** Top differentially expressed genes in HTNV-infected HUVECs (GSE133751). FDR-significant genes ($|\log_{2}\mathrm{FC}|\geq 1$, FDR <0.05) are shown. Direction: UP = upregulated; DOWN = downregulated.

Gene	log_2_FC	*p*-Value	FDR	Direction
*MX2*	6.04	$2.1\times 10^{-4}$	0.012	UP
*CXCL11*	5.18	$3.4\times 10^{-4}$	0.015	UP
*CXCL10*	5.17	$3.6\times 10^{-4}$	0.015	UP
*OASL*	4.86	$4.1\times 10^{-4}$	0.016	UP
*CMPK2*	4.73	$4.8\times 10^{-4}$	0.017	UP
*EPSTI1*	3.65	$6.2\times 10^{-4}$	0.019	UP
*DDX58*	3.51	$7.1\times 10^{-4}$	0.020	UP
*TRIM22*	2.14	$1.1\times 10^{-3}$	0.026	UP
*BLZF1*	2.08	$1.3\times 10^{-3}$	0.028	UP
*TRIM38*	1.92	$1.8\times 10^{-3}$	0.035	UP
*EIF4B*	−1.55	$1.9\times 10^{-3}$	0.041	DOWN

**Table 3:** Top 10 network centrality hubs in the hantavirus-responsive PPI network (STRING, score ≥0.4). MRS: Master Regulator Score (Equation (1)). Degree; $C_{B}$: betweenness centrality; $C_{C}$: closeness centrality. The log_2_FC values are from the GSE133751 analysis.

Gene	MRS	Degree	$C_{B}$	$C_{C}$	log_2_FC	FDR
*ISG15*	1.000	100	0.038	0.71	3.21	0.048
*IRF1*	0.952	92	0.038	0.70	2.87	0.052
*CXCL10*	0.901	86	0.039	0.69	5.17	0.015
*IFI35*	0.878	96	0.034	0.68	2.43	0.061
*STAT1*	0.887	114	0.023	0.72	2.31	0.063
*IFI44L*	0.821	88	0.031	0.67	3.44	0.044
*MX1*	0.814	84	0.033	0.66	3.18	0.049
*GBP1*	0.798	80	0.035	0.65	2.76	0.055
*ZC3HAV1*	0.782	76	0.036	0.64	2.54	0.058
*TRIM25*	0.771	74	0.034	0.63	2.21	0.067

**Table 4:** Summary of epidemiological model outcomes under four intervention scenarios. All values are from 730-day simulations with initial conditions as described in Section 3.5.7.

Scenario	Peak $I_{R}$	Peak $I_{H}$/100k	Cum. $D_{H}$/10k	Attack Rate (%)
Baseline	26.1	10.00	2.98	0.119
Rodent control	16.7	10.00	3.15	0.125
Exposure reduction	26.1	2.50	0.94	0.037
Combined	16.7	2.50	0.98	0.039

## Data Availability

All expression data used in this study are publicly available from the Gene Expression Omnibus (GSE133751). Analysis code and supplementary data files are available from the corresponding author upon reasonable request.
